# Effects of prostaglandin F_2α_ (PGF_2α_) on cell-death pathways in the bovine corpus luteum (CL)

**DOI:** 10.1186/s12917-019-2167-3

**Published:** 2019-11-21

**Authors:** Agnieszka Walentyna Jonczyk, Katarzyna Karolina Piotrowska-Tomala, Dariusz Jan Skarzynski

**Affiliations:** Department of Reproductive Immunology and Pathology, Institute of Animal Reproduction and Food Research, Polish Academy of Sciences, 10-747 Tuwima 10 St ., 10 -, 748 Olsztyn, Poland

**Keywords:** Necroptosis, RIPKs, Apoptosis, Prostaglandin F_2α_, Bovine CL

## Abstract

**Background:**

Prostaglandin F_2α_ (PGF_2α_) may differentially affect viability of luteal cells by inducing either proliferation or cell death (via apoptosis or necroptosis). The diverse effects of PGF_2α_ may depend on its local vs. systemic actions. In our study, we determined changes in expression of genes related to: (i) apoptosis: caspase (CASP) 3, CASP8, BCL2 associated X (BAX), B-cell lymphoma 2 (BCL2) and (ii) necroptosis: receptor-interacting protein kinase (RIPK) 1, RIPK3, cylindromatosis (CYLD), and mixed lineage kinase domain-like (MLKL) in the early and mid-stage corpus luteum (CL) that accompany local (intra-CL) vs. systemic (i.m.) analogue of PGF_2α_ (aPGF_2α_) actions. Cows at day 4 (n = 24) or day 10 (n = 24) of the estrous cycle were treated by injections as follows: (1) systemic saline, (2) systemic aPGF_2α_ (25 mg; Dinoprost), (3) local saline, (4) local aPGF_2α_ (2.5 mg; Dinoprost). After 4 h, CLs were collected by ovariectomy. Expression levels of mRNA and protein were investigated by RT-q PCR, Western blotting and immunohistochemistry, respectively.

**Results:**

We found that local and systemic administration of aPGF_2α_ in the early-stage CL resulted in decreased expression of CASP3 (*P* < 0.01), but *CASP8* mRNA expression was up-regulated (*P* < 0.05). However, the expression of CASP3 was up-regulated after local aPGF_2α_ treatment in the middle-stage CL, whereas systemic aPGF_2α_ administration increased both CASP3 and CASP8 expression (*P* < 0.01). Moreover, we observed that both local and systemic aPGF_2α_ injections increased RIPK1, RIPK3 and MLKL expression in the middle-stage CL (*P* < 0.05) while CYLD expression was markedly higher after i.m. aPGF_2α_ injections (*P* < 0.001). Moreover, we investigated the localization of necroptotic factors (RIPK1, RIPK3, CYLD and MLKL) in bovine CL tissue after local and systemic aPGF_2α_ injections in the bovine CL.

**Conclusion:**

Our results demonstrated for the first time that genes related to cell death pathways exhibit stage-specific responses to PGF_2α_ administration depending on its local or systemic actions. Locally-acting PGF_2α_ plays a luteoprotective role by inhibiting apoptosis and necroptosis in the early CL. Necroptosis is a potent mechanism responsible for structural CL regression during PGF_2α_-induced luteolysis in cattle.

## Background

The corpus luteum (CL) plays a crucial role in supporting pregnancy in cattle and other mammalian species, because of its production of progesterone (P_4_) [1, 2]. If pregnancy is not established, the bovine CL undergoes regression due to the action of uterine prostaglandin F_2α_ (PGF_2α_) which is released in the late luteal phase of the estrous cycle [3].

The cascade of CL regression consists of: (i) functional luteolysis (interruption of steroidogenesis), and (ii) structural luteolysis (degradation/demise of CL tissue due to cell death) [4, 5]. Until now, a large number of reports have indicated that the caspase (CASP) – dependent apoptosis (type I programmed cell death) is the principal mechanism of CL cell death during structural luteolysis in cows [6, 7]. Several mediators are involved in the regulation and control of apoptosis in the CL, among them: B-cell lymphoma 2 (BCL2), and BCL2-associated X (BAX), which belong to the bcl-2 protein family (8), and caspases (CASP) [8, 9].

Recently, Hojo et al. [10, 11] proposed that necroptosis (CASP – independent cell death pathway) is an alternative luteolytic mechanism responsible for death of luteal steroidogenic cells (LSC) and luteal endothelial cells (LEC) and for their elimination from the bovine CL during luteolysis. This process is characterized by disrupted cellular membranes with leakage of their intracellular contents and tissue damage [12, 13]. In the clasical necroptosis pathway, receptor-interacting protein kinase 1 (RIPK1) is necessary for the activation of receptor-interacting protein kinase 3 (RIPK3) [14, 15]. Moreover, the deubiquitination of RIPK1 by cylindromatosis (CYLD), a K63-specific deubiquitinating enzyme (DUB), is crucial for initiation of necroptosis and mitochondrial complex II formation [16]. In the absence of CYLD, the generation of complex II is inhibited. The activation of RIPK3 and RIPK3 substrate-mixed lineage kinase domain-like (MLKL) by its phosphorylation [17] are key steps during the execution of necroptosis [18].

In farm animals, PGF_2α_ and its analogues (aPGF_2α_) are widely used as pharmacological tools to induce luteolysis [19]. However, the newly formed CL is refractory to exogenous PGF_2α_ before day 5 of the estrous cycle. Therefore, a single PGF_2α_ treatment is ineffective for inducing luteolysis during the early luteal phase [19, 20]. Although the luteolytic action of PGF_2α_ on the regression process has been widely studied [21–23], the mechanism of CL insensitivity, the acquisition of luteolytic capacity by the CL as well as mechanisms related to its stage-specific response to PGF_2α_ all still need intensive studies. Previous studies [23–26] have suggested that the different actions of PGF_2α_ on steroidogenesis pathways, immune functions and on pro- or anti-angiogenic factors may depend on the phase of the estrous cycle: the early-stage CL (PGF_2α_-resistant) vs. middle-stage CL (PGF_2α_-responsive). However, the effects of PGF_2α_ on luteal steroidogenic cells may depend on its local, direct (autocrine/paracrine modes of action) effect or on indirect effects including several regulatory mechanisms within the female reproductive tract (e.g., endocrine action, blood flow regulation, contribution of the immune system, etc.) [27–29].

Prostaglandin F_2α_ is essential for manipulate bovine reproduction because in dairy cattle farming using of hormonal treatments are very common procedures to influence the estrous cycle. General in this study, we demonstrated the effect PGF_2α_ on new mechanism involved in the CL regression in cows (necroptosis), and that could provide new knowledge to optimize breeding methods of cows. Therefore, we intended to extend the understanding of the luteolytic process, and we hypothesised that PGF_2α_ might induce various mechanisms of cell death (differences in luteal responses) in the bovine CL depending on its peripheral and local actions during the early and mid-luteal phase of the estrous cycle. The aim of the present study was to examine the differences in expression of genes related to: (i) apoptosis (CASP3, CASP8, BAX, BCL2) and (ii) necroptosis (RIPK1, RIPK3, CYLD, MLKL) in response to intra-CL (local) or i.m. (systemic) aPGF_2α_ injections in the early- (day 4 of the estrous cycle) vs. middle-stage (day 10) bovine CL.

## Results

### Experiment 1. Changes in mRNA expression and protein concentration of CASP3, CASP8 and the ratio of BCL2 to BAX in the early- and midle-stage CL in response to local or systemic administration of aPGF_2α_

**Fig. 1:**
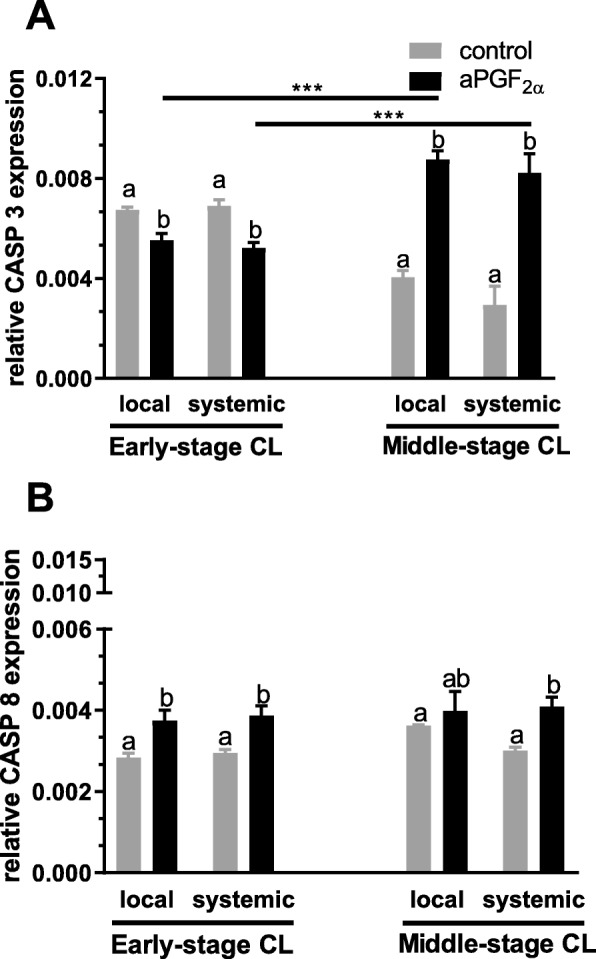
Effect of local or systemic PGF_2α_ analogue (aPGF_2α_) administration on the mRNA expression of (**a**) caspase 3 (CASP3), (**b**) caspase 8 (CASP8) in the early- and middle- stage corpora lutea (CL), respectively. The gray bars represent the control group, and the black bars represent local or systemic aPGF_2α_ administered groups. Letters ^a,b^ indicate statistical differences between all experimental groups in the early and middle- stage CL. Asteriks ^*^ indicate statistical differences between local/systemic aPGF_2α_ injected early CL vs local/systemic aPGF_2α_ injected middle-stage CL

**Fig. 2:**
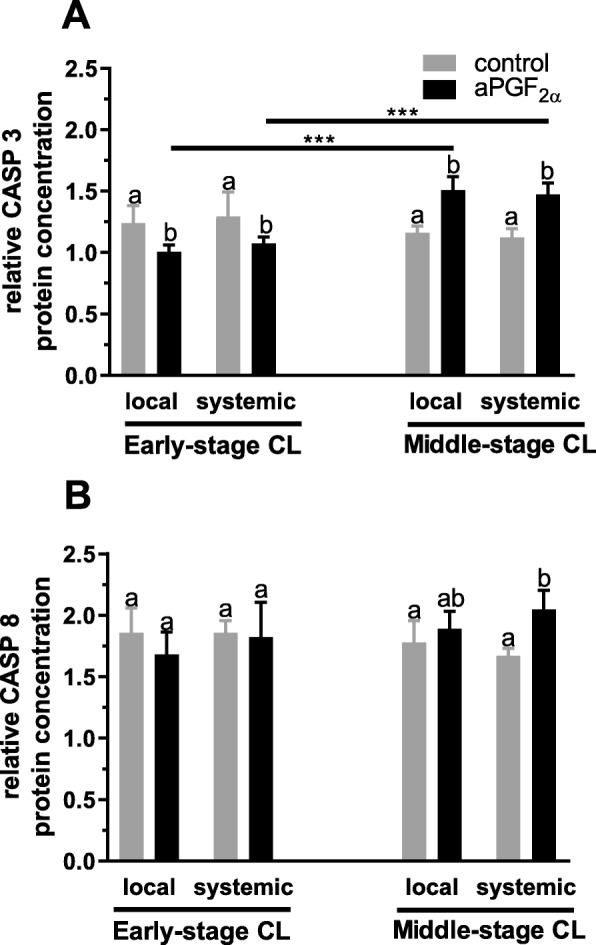
Effect of local or systemic PGF_2α_ analogue (aPGF_2α_) administration on the protein concentration of (**a**) caspase 3 (CASP3), (**b**) caspase 8 (CASP8) in the early- and middle- stage corpora lutea (CL), respectively. The gray bars represent the control group, and the black bars represent local or systemic aPGF_2α_ administered groups. Letters ^a,b^ indicate statistical differences between all experimental groups in the early and middle- stage CL. Asteriks ^*^ indicate statistical differences between local/systemic aPGF_2α_ injected early CL vs local/systemic aPGF_2α_ injected middle-stage CL

Figures 1 and 2 show the results for analysis of mRNA expression and protein concentration of CASP3 and CASP8 in the early and middle-stage bovine CL. An opposite effect of local and systemic aPGF_2α_ action was observed in the early- versus middle-stage CL (*P* < 0.0001; Fig. 1A and 2A). Local and systemic administration of aPGF_2α_ resulted in decreased mRNA expression (*P* = 0.0073, *P* = 0.0003, respectively; Fig. 1A) and protein concentration of CASP3 (*P* = 0.0021, *P* = 0.0038, respectively; Fig. 2A) in the early-stage CL, while both aPGF_2α_ treatments increased *CASP3* mRNA expression (*P* < 0.0001; Fig. 1A) and protein concentration (*P* < 0.0001; Fig. 2A) in the middle-stage CL. Hovewer, *CASP8* mRNA expression was up-regulated by local and systemic aPGF_2α_ injections in the early-stage CL (*P* = 0.0442, *P* = 0.0383, respectively; Fig. 1B), with no effect on CASP8 protein concentration (*P* = 0.4715, *P* = 0.9969, respectively; Fig. 2B). Additionally, only systemic aPGF_2α_ injection increased CASP8 mRNA expression (*P* = 0.0129; Fig. 1B) and protein concentration (*P* = 0.0152; Fig. 2B) in the middle-stage CL.

**Fig. 3:**
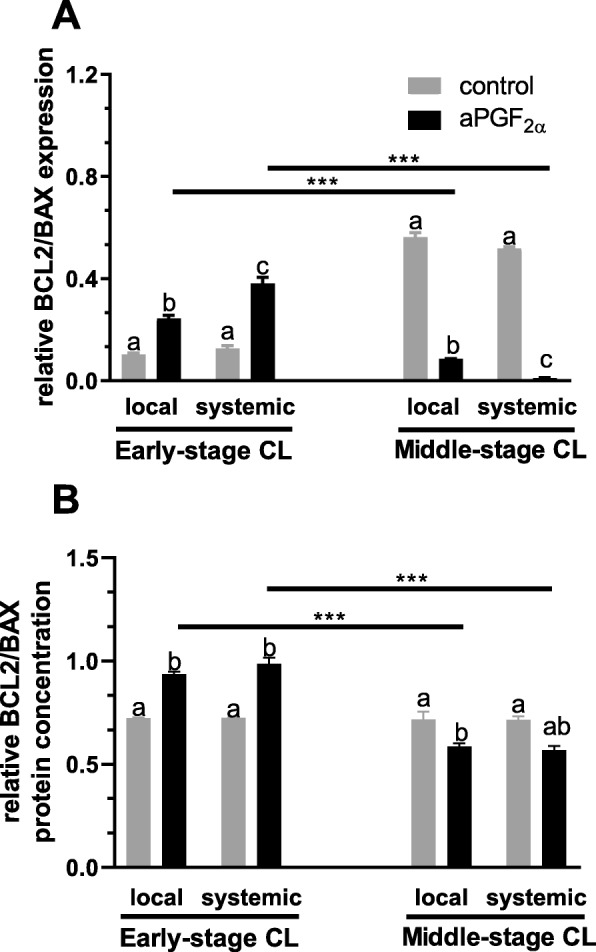
Effect of local or systemic PGF_2α_ analogue (aPGF_2α_) administration on the (**a**) the ratio of *BCL2* to *BAX* mRNA expression and (**b**) the ratio of BCL2 to BAX protein concentration levels in the early- and middle- stage corpora lutea (CL), respectively. The gray bars represent the control group, and the black bars represent local or systemic aPGF_2α_ administered groups. Letters ^a,b^ indicate statistical differences between all experimental groups in the early and middle- stage CL. Asteriks ^*^ indicate statistical differences between local/systemic aPGF_2α_ injected early CL vs local/systemic aPGF_2α_ injected middle-stage CL

Figure 3 shows the results for analysis of mRNA expression and protein concentration of the ratio of BCL2 to BAX in the early- and middle-stage CL. An opposite effect of local and systemic aPGF_2α_ action was observed in the early-stage CL versus middle-stage CL (*P* = 0.0003, *P* < 0.0001, respectively; Fig. 3A), namely local and systemic aPGF_2α_ administration increased the ratio of *BCL2* to *BAX* mRNA expression in the early-stage CL (*P* = 0.0164 and *P* < 0.0001, respectively; Fig. 3A), while both aPGF_2α_ treatment decreased its mRNA expression in the middle-stage CL (*P* < 0.0001; Fig. 3A). Comparison of local to systemic administration of aPGF_2α_ showed higher *BCL2/BAX* mRNA expression after systemic aPGF_2α_ injection in the early-stage CL (*P* = 0.0099; Fig. 5A) while systemic aPGF_2α_ action induced lower *BCL2/BAX* mRNA expression in the middle-stage CL (*P* < 0.0001; Fig. 3A). Moreover, both aPGF_2α_ action enhanced the ratio of BCL2 to BAX protein concentration (*P* < 0.0001; Fig. 3B) in the early-stage CL, while their protein concentration was down-regulated by both local and systemic aPGF_2α_ treatments in the middle-stage CL (*P* = 0.0084, *P* = 0.0244, respectively; Fig. 3B). Additionally, an opposite effect of local and systemic aPGF_2α_ action on BCL2/BAX protein concentration was observed in early- versus middle-stage CL (*P* < 0.0001; Fig. 3B).

### Experiment 2. Changes in mRNA expression and protein concentration of RIPK1, RIPK3, CYLD, MLKL in the early- and middle-stage CL in response to local or systemic administration of aPGF_2α_

**Fig. 4:**
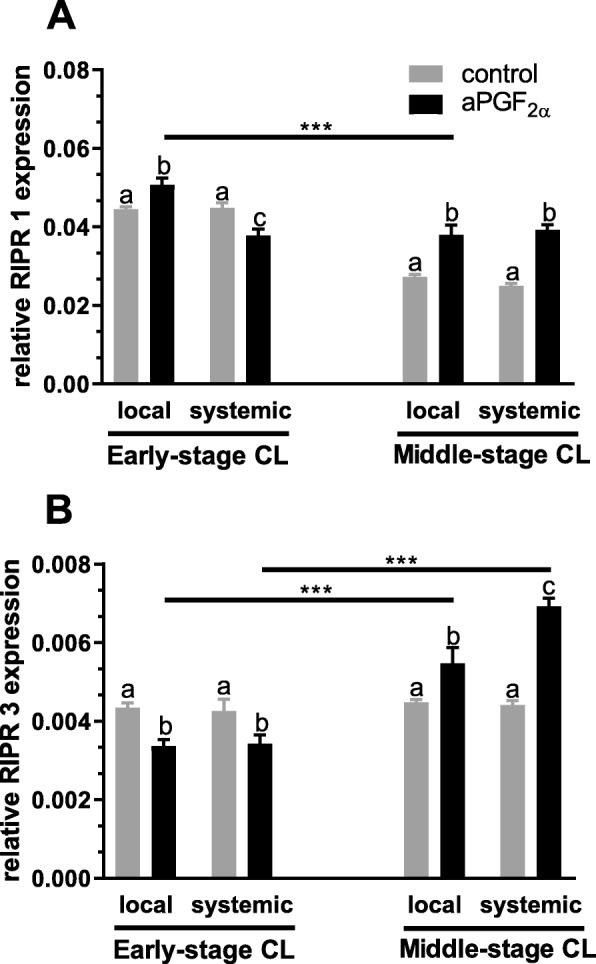
Effect of local or systemic PGF_2α_ analogue (aPGF_2α_) administration on the mRNA expression of: (**a**) *RIPK1*, (**b**) *RIPK3* in the early and middle- stage corpora lutea (CL), respectively. The gray bars represent the control group, and the black bars represent local or systemic aPGF_2α_ administered groups. Letters ^a,b,c^ indicate statistical differences between all experimental groups in the early and middle- stage CL. Asteriks ^*^ indicate statistical differences between local/systemic aPGF_2α_ injected early CL vs local/systemic aPGF_2α_ injected middle-stage CL

**Fig. 5:**
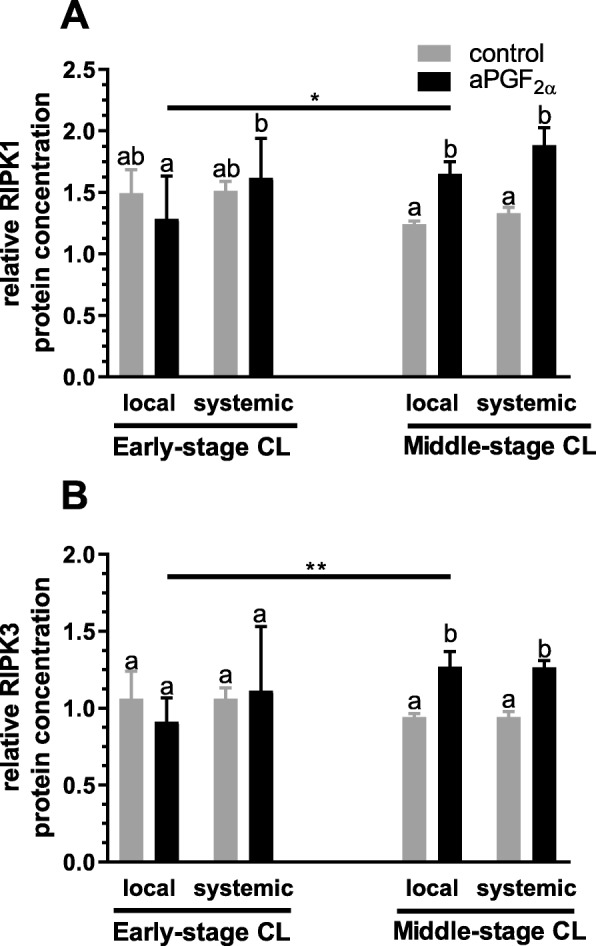
Effect of local or systemic PGF_2α_ analogue (aPGF_2α_) administration on the protein concentration of: (**a**) RIPK1, (**b**) RIPK3 in the early- and middle- stage corpora lutea (CL), respectively. The gray bars represent the control group, and the black bars represent local or systemic aPGF_2α_ administered groups. Letters ^a,b^ indicate statistical differences between all experimental groups in the early and middle- stage CL. Asteriks ^*^ indicate statistical differences between local/systemic aPGF_2α_ injected early CL vs local/systemic aPGF_2α_ injected middle-stage CL

Figures 4 and 5 show the results for analysis of mRNA expression and protein concentration of RIPK1 and RIPK3 in the early- and middle-stage bovine CL. A local aPGF_2α_ injection up-regulated *RIPK1* mRNA expression (*P* = 0.0288; Fig. 4A) but down-regulated *RIPK3* mRNA expression (*P* = 0.0130; Fig. 4B) in the early-stage CL. However, systemic aPGF_2α_ administration resulted in a decrease in *RIPK1* and *RIPK3* mRNA expression in the early-stage CL (*P* = 0.0112, *P* = 0.0407; Fig. 4A and B). Furthermore, both local and systemic aPGF_2α_ injections increased the mRNA expression of *RIPK1*(*P* = 0.0002, *P* < 0.0001, respectively; Fig. 4A) and *RIPK3* (*P* = 0.0124, *P* < 0.0001, respectively; Fig. 4B) in the middle-stage CL. Additionally, we obserwed higher *RIPK1* mRNA expression in the early-stage CL after local aPGF_2α_ treatment compared with that in middle-stage CL(*P* < 0.0001; Fig. 4A). However, both routes of aPGF_2α_ treatment had an opposite effect in *RIPK3* mRNA expression in the early-stage CL compared with that action in the middle-stage CL (*P* < 0.0001; Fig. 4B). Moreover, both aPGF_2α_ injections increased protein concentration of RIPK1(*P* = 0.0239, *P* < 0.0019, respectively; Fig. 5A) and RIPK3 (*P* = 0.0263, *P* = 0.0279, respectively; Fig. 5B) in the middle-stage CL. Only local aPGF_2α_ action had an opposite effect on RIPK1 (*P* = 0.0128; Fig. 5A) and RIPK3 (*P* = 0.0068; Fig. 5B) protein concentration observed in the early- versus middle-stage CL. Additionally, comparison of local and systemic administration of aPGF_2α_ showed differences in *RIPK1* mRNA expression level in the early-stage CL (*P* < 0.0001; Fig. 5A). Moreover *RIPK3* mRNA expression was greater in the middle-stage CL in response to systemic aPGF_2α_ injection compared with the local treatment route (*P* = 0.0003; Fig. 5B).

**Fig. 6:**
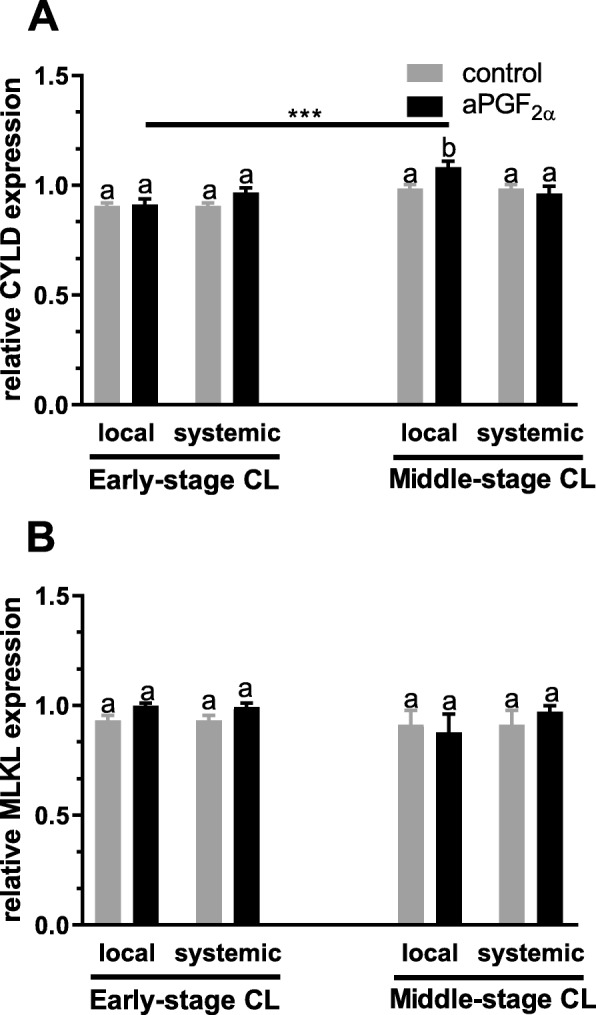
Effect of local or systemic PGF_2α_ analogue (aPGF_2α_) administration on the mRNA expression of: (**a**) *CYLD*, (**b**) *MLKL* in the early - and middle-stage corpora lutea (CL), respectively. The gray bars represent the control group, and the black bars represent local or systemic aPGF_2α_ administered groups. Letters ^a,b^ indicate statistical differences between all experimental groups in the early and middle- stage CL. Asteriks ^*^ indicate statistical differences between local/systemic aPGF_2α_ injected early CL vs local/systemic aPGF_2α_ injected middle-stage CL

**Fig. 7:**
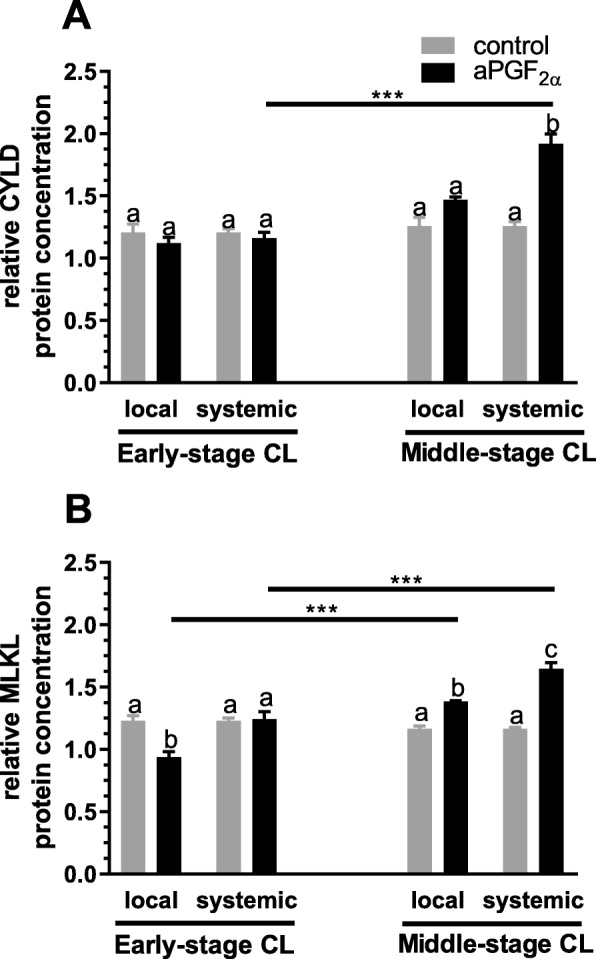
Effect of local or systemic PGF_2α_ analogue (aPGF_2α_) administration on the protein concentration of: (**a**) CYLD, (**b**) MLKL in the early- and middle-stage corpora lutea (CL), respectively. The gray bars represent the control group, and the black bars represent local or systemic aPGF_2α_ administered groups. Letters ^a,b,c^ indicate statistical differences between all experimental groups in the early and middle- stage CL. Asteriks ^*^ indicate statistical differences between local/systemic aPGF_2α_ injected early CL vs local/systemic aPGF_2α_ injected middle-stage CL

Figures 6 and 7 show the results for analysis of mRNA expression and protein concentration of CYLD and MLKL in the early- and middle-stage bovine CL. Only local aPGF_2α_ administration up-regulated *CYLD* mRNA expression in the middle-stage CL (*P* = 0.0127; Fig. 6A). However, only systemic aPGF_2α_ injection enhanced its protein concentration in the middle-stage CL (*P* < 0.0001; Fig. 7A). Furthermore, local aPGF_2α_ injection induced down-regulation of MLKL protein concentration in the early-stage CL (*P* = 0.0002; Fig. 7B). On the other hand, local and systemic aPGF_2α_ treatments increased its protein concentration in the middle-stage CL (*P* = 0.0033, *P* < 0.0001; Fig. 7B); however, this effect was greater when aPGF_2α_ was injected systematically (P < 0.0001; Fig. 7B). Moreover, an opposite effect of local and systemic aPGF_2α_ action on MLKL protein concentration was observed in the early- versus middle-stage CL (*P* < 0.0001; Fig. 7B).

### Experiment 3. Immunohistochemistry localization and changes in intensities of RIPK1, RIPK3, CYLD, MLKL in the early- and middle-stage CL in response to local or systemic administration of aPGF_2α_

**Fig. 8:**
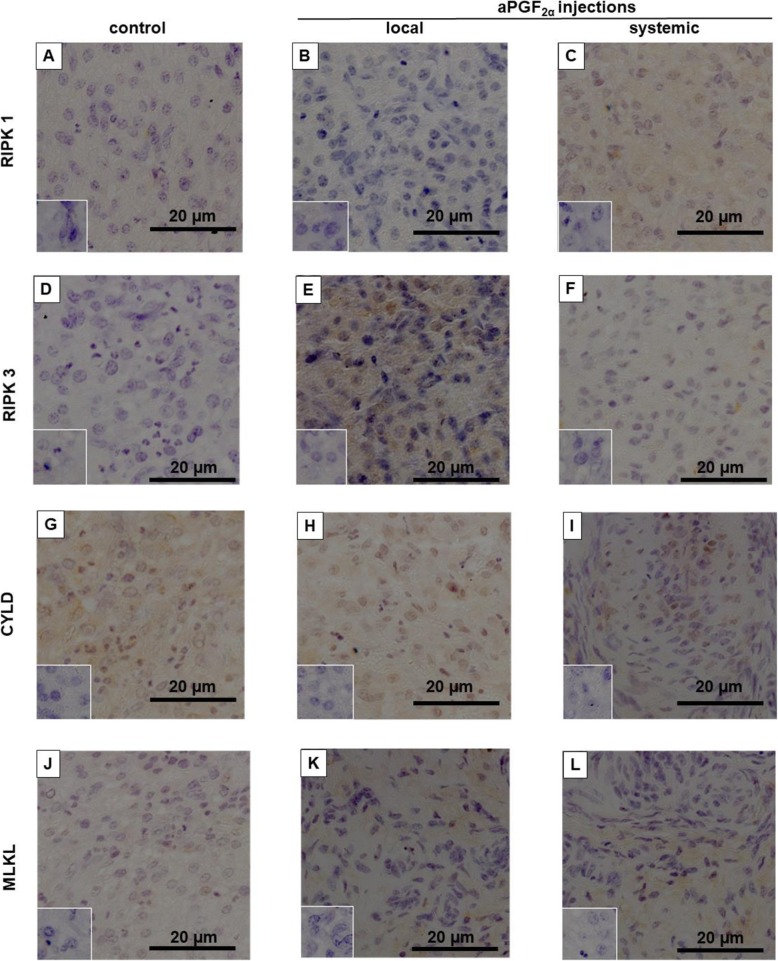
Representative section of images of localization of: (**a, b, c**) RIPK1, (**d, e, f**) RIPK3, (**g, h, i**) CYLD and (**j, k, l**) MLKL protein in the bovine early-stage corpora lutea (CL) at 4 h after local or systemic PGF_2α_ analogue (aPGF_2α_) administration. Each small window shows a negative control stained with normal rabbit IgG instead of primary antibody. Positive immunohistochemistry staining was assessed as brown staining. Bar = 20 μm

**Fig. 9:**
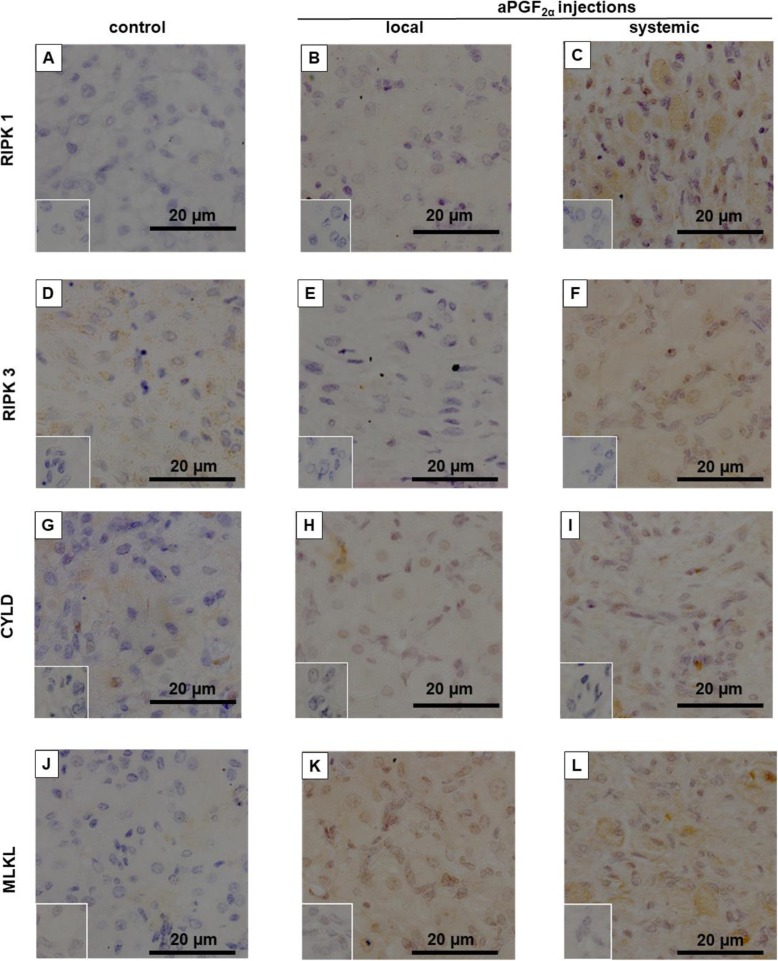
Representative section of images of localization of: (**a, b, c**) RIPK1, (**d, e, f**) RIPK3, (**g, h, i**) CYLD and (**j, k, l**) MLKL protein in the bovine middle-stage corpora lutea (CL) at 4 h after local or systemic PGF_2α_ analogue (aPGF_2α_) administration. Each small window shows a negative control stained with normal rabbit IgG instead of primary antibody. Positive immunohistochemistry staining was assessed as brown staining. Bar = 20 μm

In another set of studies, we investigated the localization of RIPK1, RIPK3, CYLD and MLKL in bovine CL tissue after local and systemic aPGF_2α_ treatment by immunohistochemistry. Representative sections of images are shown in Fig. 8 (early-stage CL) and Fig. 9 (middle-stage CL).

**Fig. 10:**
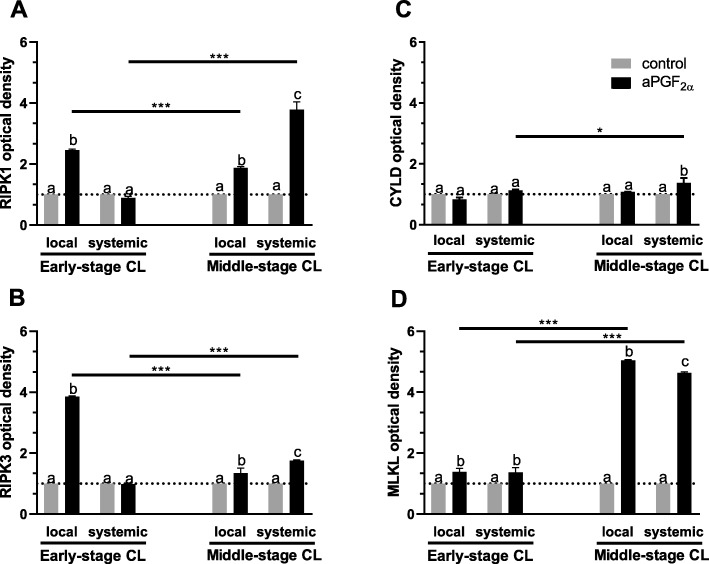
The arithmetic means of intensities of: (**a**) RIPK1, (**b**) RIPK3, (**c**) CYLD and (**d**) MLKL in the bovine early- and middle-stage corpora lutea (CL) after local or systemic PGF_2α_ analogue (aPGF_2α_) administration. The gray bars represent the control group, and the black bars represent local or systemic aPGF_2α_ administered groups. Letters ^a,b,c^ indicate statistical differences between all experimental groups in the early and middle- stage CL. Asteriks ^*^ indicate statistical differences between local/systemic aPGF_2α_ injected early-stage CL vs local/systemic aPGF_2α_ injected middle-stage CL

Figure 10 shows the arithmetic means of intensities of RIPK1, RIPK3, CYLD and MLKL in the early- and middle-stage CL after local or systemic aPGF_2α_ treatment. The intensity of RIPK1 was up-regulated after local aPGF_2α_ injection in the early-stage CL (*P* < 0.0001; Fig. 10A). Moreover, local and systemic aPGF_2α_ treatments increased its intensity in the middle-stage CL (*P* < 0.0001; Fig. 10A); however, this effect was greater when aPGF_2α_ was injected systematically (*P* < 0.0001; Fig. 10A). Additionally, we observed higher intensity of RIPK1 in the middle-stage CL after systemic aPGF_2α_ treatment compared with that in the early-stage CL (*P* = 0.0009; Fig. 10A). Furthermore, local aPGF_2α_ injection increased the intensity of RIPK3 in the early- and middle-stage CL compared to the control group (*P* < 0.0001, *P* = 0.0030, respectively; Fig. 10B); while systemic aPGF_2α_ treatment up-regulated the intensity of RIPK3 only in the middle-stage CL compared to the control group (*P* < 0.0001; Fig. 10B). Comparison of local to systemic administration of aPGF_2α_ showed differences in RIPK3 intensity level in early- and middle-stage CL (*P* < 0.0001; Fig. 10B). Moreover, we observed higher RIPK3 intensity after local aPGF_2α_ injection in the early-stage CL compare with that after local aPGF_2α_ administration in the middle-stage CL (*P* < 0.0001; Fig. 10B) while RIPK3 intensity was higher after systemic aPGF_2α_ treatment in the middle-stage CL compare to systemic injection in the early-stage CL (*P* < 0.0001; Fig. 10B). Systemic aPGF_2α_ administration increased CYLD intensity (*P* = 0.0012, Fig. 10C). Comparison of systemic action of aPGF_2α_ in both stages showed higher intensity of CYLD in the middle-stage CL (*P* = 0.0274; Fig. 10C). Moreover we obserwed higher CYLD intensity after systemic aPGF_2α_ action compare to its intensity after local aPGF_2α_ injection in the middle-stage CL (*P* = 0.0052; Fig. 10C). Futhermore, local and systemic aPGF_2α_ treatments enhanced intensity of MLKL in the early- (*P* = 0.0048, *P* = 0.0081, respectively; Fig. 10D) and middle-stage CL (*P* < 0.0001; Fig. 10D). Comparison of local and systemic administration of aPGF_2α_ showed differences in MLKL intensity in the middle-stage CL (*P* = 0.0016; Fig. 10D). Additionally, we observed higher MLKL intensity after local and systemic aPGF_2α_ injection in the middle-stage CL compare with that after both aPGF_2α_ administration in the early-stage CL (*P* < 0.0001; Fig. 10D).

## Discussion

Prostaglandin F_2α_ is essential for manipulate bovine reproduction because in dairy cattle farming using of hormonal treatments are very common procedures to influence the estrous cycle. Until now, there have been no reports indicating a clear difference in PGF_2α_ effects on cell-death mechanisms in the bovine CL with regard to its auto- or para-crine (local administration into the CL) vs. endocrine actions (systemic administration). Moreover, we confirmed in our previous in vivo and in vitro studies that during PGF_2α_-induced regression of the bovine CL, luteal cells are eliminated not only by apoptosis but also by programmed necrosis (RIPK-dependent necroptosis) [10, 11]. Futhermore, this is the first study describing details of a necroptotic pathway during PGF_2α_-induced luteal regression, showing up-regulation of CYLD and MLKL expression after PGF_2α_ administration by both administration routes in the middle-stage CL. Therefore, we assume that better understanding of the effect of PGF_2α_ on new mechanism involved in the CL regression in cows (necroptosis) may improve knowledge to optimize breeding methods of cows.

Prostaglandin F_2α_ through its potent mediators plays a crucial role in regulation of the luteolytic cascade [21, 29], modulating numerous proteins associated with cell survival and cell death in different species [30]. It is well known that many factors are involved in PGF_2α_-induced luteolysis in cattle, including proinflammatory cytokines such as tumor necrosis factor α (TNF), interferon gamma (IFNG), Fas ligand (FASLG) [6, 31], endothelin 1 (EDN1) [32] and nitric oxide (NO) [28]. Moreover, communication between luteal and non-luteal cells is required for development and regression of the bovine CL [27, 33]. On the other hand, the lack of luteolytic effects of PGF_2α_ in the bovine early CL may be associated with the absence of a well-established vascular system, despite the intensive angiogenesis occurring at this time [22, 34]. The early bovine CL is refractory to luteolytic actions of PGF_2α_ in spite of the presence of PGF_2α_ receptors [20]. However, the mechanism of insensitivity and acquisition to sensitivity of the CL to PGF_2α_ is still not fully understood [23, 24, 26, 35].

The role of PGF_2α_ in activating apoptotic signaling cascades in the CL during PGF_2α_- induced luteolysis has been previously examined [5]. The ratio of *BCL2* to *BAX* expression levels is essential for cell survival or death [36–38]. In our study, we observed that the ratio of *BCL2* to *BAX* mRNA expression levels was higher after systemic aPGF_2α_ treatment compared to its local injection in the early-stage CL. In contrast, in the middle-stage CL this ratio of mRNA expression was markedly decreased after systemic PGF_2α_ injection compared to its local administration. These findings suggest that PGF_2α_ actions in the bovine CL depend on specific mediators participating in the progress of apoptosis by increasing BAX expression. Yadav et al. [39] found increased BAX and a constant mRNA and protein expressionof BCL2 in buffalo in the middle-stage CL 4 h after PGF_2α_ treatment. In contrast, Kleim et al. [40] reported increased mRNA expression of *BAX* at 24 h after induced luteolysis, but in that study a different PGF_2α_ analogue was used, which might explain the later increase in expression level.

In our study, we noticed that both PGF_2α_ administration routes decreased CASP3 expression in the early CL, while increasing its expression in the middle-stage CL. Moreover, we showed that induction of *CASP8* mRNA expression was responsive only to systemic PGF_2α_ administration in the middle-stage CL. Additionally, there were no significant differences in *CASP3* and *CASP8* mRNA expression and protein concentration between local vs. systemic treatments with aPGF_2α_ in both the early and middle-stage CL. These results correspond with the findings of other studies which reported that induction of *CASP3* is an important factor in luteolysis, as well as the increase in *CASP3* mRNA expression that occurs during PGF_2α_-induced luteolysis in the CL of different species [5, 8, 40].

Furthermore, one of the apoptosis initiators is CASP8 [41]. In the present study, we showed that induction of CASP8 mRNA expression and protein concentration was responsive only to systemic administration of PGF_2α_ in the middle-stage CL. Therefore, we conclude that PGF_2α_ is most potent as a luteolytic factor when it reaches the CL through the blood vasculature. It is well known that activated CASP8 promotes the apoptotic cascade by cleaving CASP3 [42]. Moreover, during the process of apoptosis accomplished via the mitochondrial pathway, active CASP8 regulates the binding of pro-apoptotic BAX to mitochondria and inhibits their connection with the antiapoptotic BCL2.

On the other hand, our present results indicate that either local or systemic action of PGF_2α_ suppressed apoptotic signals via CASP3, and in parallel by affecting BCL2 and BAX expression in the early-stage CL. Therefore, PGF_2α_ may exert an anti-apoptotic action on bovine luteal cells, thus playing a luteoprotective role in the early-stage CL. It is important to realise that CASP8 may be bound by some death effector domain (DED)-containing proteins such as cellular FLICE-like inhibitory protein (c-FLIP), which can inhibit apoptosis [43]. Our observations may explain why up-regulation of *CASP8* mRNA expression in the early-stage CL was not followed by increased mRNA expression of effector *CASP3*. Importantly, the CASP8-cFLIP complex prevents not only apoptosis but also RIPK-induced necroptosis [44].

During the process of structural CL regression, not only apoptosis should be taken into consideration. Recently, Hojo et al. [10, 11] demonstrated that i.m. administration of PGF_2α_ up-regulated both RIPK1 and RIPK3 expression in bovine CL cells and tissue in vivo and in vitro. In contrast to apoptosis, necroptosis occurs in the absence of CASPs activity (CASPs-independent programmed cell death). In our study, we investigated the regulatory mechanism of RIPK expression to clarify the mechanisms of necroptosis in the early and middle-stage CL in response to the local and systemic actions of PGF_2α_. We reported that expression of RIPK1 and RIPK3 mRNA and protein concentration was elevated after local and systemic PGF_2α_ injections in the middle-stage CL. Moreover, it is clearly confirmed by immunohistochemistry analysis showing that intensities of RIPK1 and RIPK3 were up-regulated by both PGF_2α_ treatments in the middle-stage CL. Therefore our results indicate that RIPK-dependent necroptosis is involved in aPGF_2α_-induced CL regression. Interestingly, we observed that systemic administration of PGF_2α_ markedly up-regulated *RIPK3* mRNA expression compared to its local action in the middle-stage CL. Based on the above results, we confirmed that PGF_2α_ is a crucial luteolytic factor when administered systemically and the stimulatory effect of PGF_2α_ on RIPKs expression may depend on different mediators or upon cell composition and cell contacts. Moreover, unlike apoptosis, necroptosis induces a more marked immune response that may function as a defensive mechanism [45]. A variety of cytokines produced by an increasing variety of local immune cells may be involved in the induction of luteal cell death processes in the bovine CL [3, 46].

In our study, systemic PGF_2α_ injection may inhibit the necroptotic pathway by decreasing *RIPK1* and *RIPK3* mRNA expression in the early-stage CL. Additionally, we observed that local injection of PGF_2α_ down-regulated *RIPK3* mRNA expression, while oppositely affecting *RIPK1* mRNA expression in the early-stage CL. Therefore, we suggest that formation of necrosomes does not occur in the early-stage CL. It has been reported that RIPK1 is a crucial mediator for RIPK3 activity and serves as a key mediator of cell death [18]. Therefore, we suspected that PGF_2α_ through suppression of the death pathway in luteal cells may play a luteoprotective role in the early-stage CL. Bowolaksono et al. [47] suggested that PGF_2α_ produced by bovine luteal cells inhibits apoptosis via stimulation of P_4_ in these cells, therefore luteal PGF_2α_ is thought to be a luteoprotective factor [48].

To our knowledge, this is the first report showing the expression of CYLD and MLKL in the bovine CL. Interestingly, we observed higher protein concentration of CYLD and MLKL after systemic aPGF_2α_ treatment compared to its local administration in the middle-stage CL. Additionally, immunohistochemistry analysis confirmed that intensity of CYLD was higher after systemic PGF_2α_ treatment, while both administration of PGF_2α_ affect intensity of MLKL in the middle-stage CL. It is known that CYLD is a key factor regulating cell survival and cell death, in a variety of ways including CASP8-mediated cell apoptosis and CASP8-independent cell necrosis [12, 49]. Moreover, MLKL is so far the most potent downstream effector of necroptosis that has been identified [50, 51]. The deubiquitination of RIPK1 by CYLD is critical for the activation of necroptosis and complex II formation [16]. In our study, we observed an increase in expression of the above-mentioned RIPKs and CYLD or MLKL, suggesting the activation of necroptosis and the formation of necrosomes during PGF_2α_-induced luteolysis. The above results are in agreement with previous studies carried out on different models based on selected human immune system cells [52] or embryonic fibroblasts from RIP3 knockout mice [53]. These authors emphasized the role of RIPK1, RIPK3 and MLKL as principal markers of TNF triggered necroptosis. Interestingly, in the present study mRNA expression of *RIPK3* and the protein expression of CYLD and MLKL were higher after systemic administration of PGF_2α_ compared to its local effect in the middle-stage CL. These findings may indicate that the systemic effect of PGF_2α_ on the mechanism of cell death in the CL is more effective, depending on several auto/paracrine mediators activating luteolytic mechanisms, upon cell type and on cell-to-cell contact [33], and participation of the vascular system [22, 34, 54]. Therefore, we should take into consideration the fact that the distribution of capillaries is different during luteal development and regression [24, 54].

## Conclusion

In conclusion, we have confirmed that PGF_2α_ differentially modulates the expression of genes involved in apoptosis and necroptosis depending on the route of its administration (local vs. systemic), while local PGF_2α_ plays a luteoprotective role by inhibiting necroptosis and apoptosis pathways in the early-stage CL. We confirmed that RIPK-dependent necroptosis is a potent mechanism involved in structural CL regression during PGF_2α_-induced luteolysis in cattle. Interestingly, the mechanism of the necroptotic pathway was evidently more affected by systemic PGF_2α_ actions compared to its local impact during PGF_2α_-induced regression in the middle-stage CL, confirming that PGF_2α_ influences CL function through auto/paracrine mediators.

## Methods

### Ethical authorization

The present authors ensured that their manuscript reported adheres to the arrive guidelines for the reporting of animal experiments. This statement address to their manuscript that these guidelines were followed: EU Directive of the European Parliament and the Council on the protection of animals used for scientific purposes (22 September 2010; no 2010/63/EU), the Polish Parliament Act on Animal Protection (21 August 1997, Dz.U. 1997 No 111 poz. 724) with further updates – the Polish Parliament Act on the protection of animals used for scientific or educational purposes (15 January 2015, Dz.U. 2015 pos. 266). All animal procedures were designed to avoid or minimize discomfort, distress and pain to the animals, moreover, were reviewed and accepted following the guidelines of the Local Ethics Committee for Experiments on Animals in Olsztyn, Poland (Approval no 23/2012/N).

### Animals and treatments

For the present study, 48 healthy, cycling Polish Holstein-Friesian cows from a local commercial dairy farm were used. The history of the cows and the structure of the farms were investigated by a questionnaire for the owners. Written owner consent was available through farm manager. This study was conducted from May 2018 to December 2018. The cows were bred by artificial insemination with a standard, routine protocol. The farm was monitored by trained veterinary and nutrition consultants and was free of Bovine Herpesvirus Type 1 (BHV1), Bovine Viral Diarrhea-Mucosal Disease (BVD/MD virus), Enzootic bovine leukosis (EBL) and tuberculosis. The experiment was performed in a group of non-pregnant cows (*n* = 48; 612 ± 97 kg; 3 to 5 parities; aged 5–7 years) and that were considered for culling because of their low milk production. The experimental cows were housed in an indoor facility in free-stall barns, were milked on a 12 h cycle, and fed with a TMR to meet the nutritional requirements of milking cows (15–20 kg/day) with ad libitum access to water and a salt-based mineral supplement. Prior to the experiment, an experienced veterinarian confirmed the absence of reproductive tract disorders by an ultrasonographic visualization (USG) per rectum with a 7.5 MHz linear array transducer (MyLab 30VET Gold Colour Doppler Diagnostic Ultrasound System, ESOATE Pie Medica, Genoa, Italy). Moreover, all the experimental cows underwent a general clinical examination in which rectal temperature (38.0–39.2 °C), general attitude (healthy), respiratory rates (27–30 breaths per minute), heart rates (60–82 beats per minute) and BCS (3.0 ± 0.5) were determined. The estrous cycle was synchronized in all cows by two injections of aPGF_2α_ (Dinoprost, 25 mg/5 ml; Dinolytic; Zoetis, Poland) with an 11-days interval, as reported previously [55]. Follicular development and structural changes of the CL during the entire estrous cycle were monitored using transrectal USG, and visible signs of estrus (i.e., vaginal mucus and standing behavior) were taken as its confirmation. The onset of estrus was considered as day 0 of the estrous cycle. Additionally, the stage of the estrous cycle was established by P_4_ concentrations in blood plasma samples collected from the coccygeal vessels using radioimmunoassay (RIA). The concentration of P_4_ was 0.38 ± 0.09 ng/ml (mean ± SEM**)** in blood samples collected during estrus (day 0 of the estrous cycle). After our in vivo study the experimental cows (n = 48) were slaughtered in local abbatoir due to farmer’s breeding and management program.

### In vivo study design

**Fig. 11:**
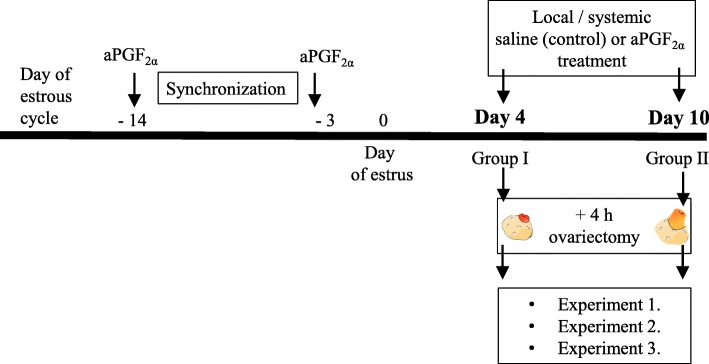
Schematic diagram of the study design. Cows were synchronized via two injections of aPGF_2α_ administered with an 11-days interval, starting on protocol day − 14. The onset of estrus was considered as day 0 of the estrous cycle. On day 4 or 10 respectively, the cows received an local injection of saline/ aPGF_2α_ (2.5 mg Dinoprost/0.5 ml) or systemic injection of saline/ aPGF_2α_ (25 mg Dinoprost/5 ml). At 4 h after treatment the cows were ovariectomized. CLs were collected for gene and protein expression

The cows were divided into two groups depending on the phase of the estrous cycle: group I (early luteal phase, day 4 of the estrous cycle; *n* = 24) and group II (day 10, mid-luteal phase; n = 24). The concentration of P_4_ was 2.98 ± 0.46 ng/ml or 9.54 ± 0.28 ng/ml (mean ± SEM) in blood samples collected from cows in group I or II, respectively. Afterwards, the cows were treated as follows: (1) i.m. (systemic) sterile 0.9% saline solution injection (control; *n* = 6), (2) systemic aPGF_2α_ injection (25 mg/5 ml Dinoprost; Dinolytic, Zoetis, Poland; n = 6), (3) intra-CL (local) saline injection (control; n = 6), and (4) local aPGF_2α_ injection (2.5 mg/0.5 ml Dinoprost; n = 6). Figure 11 shows in vivo study design. The dose of aPGF_2α_ for intra-CL injection was established in our previous study [56]. The time of injections of saline solution or aPGF_2α_ was defined as hour ‘0’. Before intra-CL injections, the animals were premedicated with xylazine (i.m. 25–30 mg/animal; Xylavet 2%; ScanVet, Poland). Then, the cows were anesthetized via an epidural block using 4 ml of 2% procaine hydrochloride (Polocainum Hydrochloricum; Biowet Drwalew, Poland). Then, intra-CL injections were administered under ultrasound guidance through a sterile 1.25 × 50 mm (18 G × 2″). Ovum Pick-up disposable veterinary injection needle (BOVIVET, Poland). The transducer and needle guide were coated with a sterile lubricant (Medicum, Poland) and positioned within the vagina. We perform intra-CL injection with USG guided ovum pick-up system in cattle. The ovary bearing the CL was positioned rectally to visualize it. The needle was then passed through the vaginal wall, and the aPGF_2α_ or saline was injected directly into the CL. Each disposable catheter was filled with 0,5 mL saline/ dinoprost. Moreover, the injected substance was observed by USG as a white shade on the monitor and it was seen to diffuse within the CL. Four hours after each treatment, the ovaries with CL were collected by colpotomy using a Hauptner’s efeminator (Hauptner & Herberholz GmbH & Co. KG, Solingen, Germany). Ovary collection was described previously by Piotrowska et al. [57]. To avoid or minimize discomfort, distress and pain to the animals during the in vivo study, all experimental cows were kept in a barn as the separate group. Moreover, during the experimental day, the cows were fed with grass hay and were given free access to water. After experimental procedures cows were put into 24 h observation and quarantine and return to farmer’s breeding and management program. After that cows were slaughtered in local abbatoir due to farmer’s breeding and management program.

Each CL tissue was divided into three parts. The tissue was immediately placed into a 1.5 ml microcentrifuge tube containing either 1 ml RNALater (#R0901, Sigma Aldrich, Germany) or which was empty, immediately homogenized, and stored at − 80 °C. mRNA and protein expression of apoptosis - or necroptosis-related factors in CL tissues after local and systemic injections of saline or aPGF_2α_ were examined by RT-qPCR and western blotting, respectively. For immunohistochemistry, the third part of the CL tissue was fixed in 4% (vol/vol) neutral formalin (pH 7.4) for 20–24 h and then embedded in paraffin wax.

#### RNA extraction and cDNA production

Total RNA was extracted from CL tissues (40 ± 5 mg) using the Total RNA Mini (#031–100, A&A Biotechnology, Poland) according to the manufacturer’s instructions. The content and purity of RNA was assessed on a NanoDrop 1000 (Thermo Fisher Scientific, ND-1000, Wilmington, DE, USA). The 260/280 absorbance ratio for all samples was approx. 2.0, and the 260/230 absorbance ratio ranged between 1.8–2.2. Then, 1 μg RNA was reverse-transcribed into cDNA using a QuantiTect Reverse Transcription Kit (#205311, Qiagen, Germany) according to the manufacturer’s instructions. The cDNA was stored at − 20 °C until Reverse transcriptional PCR or RT-q PCR was carried out.

#### RT-qPCR

RT-qPCR assays were performed in an ABI 7900 HT sequence detection system using SYBR Green PCR master mix (Applied Biosystems, Foster City, CA, USA). For the examined genes, samples (*n* = 48) were run in duplicates. Primer sequences used for determination of *CASP3*, *CASP8*, *BAX*, *BCL2*, *RIPK1*, *RIPK3*, *CYLD*, *MLKL*, glyceraldehyde-3-phosphate dehydrogenase (*GAPDH*), actin beta (*ACTB*) and 18S ribosomal RNA (*RN18S*) mRNA expression are detailed in Table 1. All primers were designed using Primer-BLAST and synthesized by Sigma (Custom Oligos Sigma Aldrich). The stability of the reference genes was determined in the NormFinder program as previously described by Andersen et al. [58]. Gene expression data are expressed relative to the best combination of two housekeeping genes and are presented as arbitrary units. In our study gene expression is expressed as a ratio of target genes to *ACTB/RN18S1*. Total reaction volume was 10 μl containing: 3 μl cDNA (10 ng), 1 μl forward and reverse primers each (500 nM) and 5 μl SYBR Green PCR master-mix. RT-qPCR was carried out as follows: initial denaturation (10 min at 95 °C), followed by 45 cycles of denaturation (15 s at 95 °C) and annealing (1 min at 60 °C). After each PCR reaction, melting curves were obtained by stepwise increases in temperature from 60 °C to 95 °C to ensure single product amplification. Specificity of the product was confirmed by electrophoresis on 2% agarose gel. RT-qPCR results were analyzed using the method described by Zhao & Fernald [59].

**Table 1:** Sequences for primers and accession numbers for genes

Gene name	Sequence of nucleotide	GenBank Accession No.	PCR products size
CASP3	5′ TGGTGCTGAGGATGACATGG ‘3 5′ GAGCCTGTGAGCGTGCTTTT’3	NM_001077840.1	163 bp
CASP8	5′ CTGAGAGAAGAGGCCCGTGA ‘3 5′ CCCGGCTTAGGAACTTGAGG ‘3	DQ319070.1	173 bp
BAX	5′ GTGCCCGAGTTGATCAGGAC ‘3 5′ CCATGTGGGTGTCCCAAAGT ‘3	U92569.1	126 bp
BCL2	5′ GAGTTCGGAGGGGTCATGTG ‘3 5′ GCCTTCAGAGACAGCCAGGA ‘3	U92434.1	203 bp
RIPK1	5′ GCAATAGCTCCAAGCAGGTC ‘3 5′ TGTGCAGCAGGAAGTCATTC ‘3	NM_001035012	148 bp
RIPK3	5′ CCAGAGAGAGCAGGTTCCAC ‘3 5′ AATCAGGCGGTTGTTGTTTC ‘3	NM_001101884.2	219 bp
CYLD	5′ GCAATGCTCAAGTCCACCT ‘3 5′ CGTGTCTCCAGTCCCAGTC ‘3	XM_015475764.2	96 bp
MLKL	5′ ACTTCCATCAGCCGACAAAC ‘3 5′ CTCCCAGAGGACAATTCCAA ‘3	XM_024978879.1	144 bp
GAPDH	5′ CACCCTCAAGATTGTCAGCA ‘3 5′ GGTCATAAGTCCCTCCACGA ‘3	BC102589	103 bp
ACTB	5′ GAGGATCTTCATGAGGTAGTCTGTCAGG ‘3 5′ CAACTGGGACGACATGGAGAAGATCTGGCA ‘3	AY141970	349 bp
RN18S1	5′ AAGTCTTTGGGTTCCGGG ‘3 5’GGACATCTAAGGGCATCACA’3	AF176811	365 bp

### Western blotting

Protein expression levels for CASP3, CASP8, BAX, BCL2, RIPK1, RIPK3, CYLD and MLKL and ACTB in the CL tissues (*n* = 48, each sample weight 100 mg) were determined by Western blotting as previously described [10]. Specific antibodies are described in detail in Table 2. Protocols for overnight incubation were used following dilution of each antibody at 4 °C (Table 2). Subsequently, membranes were incubated with a 1:20,000 dilution of secondary polyclonal anti-rabbit IgG or anti-mouse IgG alkaline phosphatase-conjugated antibodies (#S3687, #S3562, Sigma Aldrich, Germany) for 1.5 h at room temperature (RT). Immune complexes were detected using the alkaline phosphatase visualization procedure. Each sample was checked to evaluate the intensity of immunological reactions by measuring the optical density in the defined area with computerized densitometry via NIH Image (National Institutes of Health, Bethesda, MD, USA). The protein concentration profiles are presented in arbitrary units as the ratio of the test proteins to the reference protein – ACTB. Representative western blot bands for CASP3, CASP8, BAX, BCL2, RIPK1, RIPK3, CYLD and MLKL and ACTB are shown in Additional files 1 and 2.

**Table 2:** Specific antibodies used for Western immunoblotting and Immmunohistochemistry

Antibodies	Clone	Biological source	Commercial source	Dilution for WB	Dilution for IHC
Anti-ACTB	monoclonal	mouse	Sigma, #A2228	1:10.000	
Anti-CASP3	polyclonal	rabbit	Abcam, ab44976	1:500	
Anti-CASP8	monoclonal	rabbit	Abcam, ab108333	1:1.000	
Anti-BAX	polyclonal	rabbit	Abcam, ab53154	1:500	
Anti-BCL2	polyclonal	rabbit	Abcam, ab59348	1:500	
Anti-RIPK1	polyclonal	rabbit	Sigma, #SAB3500420	1:1.000	1:200
Anti-RIPK3	polyclonal	rabbit	Sigma, #SAB2102009	1:1.000	1:100
Anti-CYLD	polyclonal	rabbit	Abcam, 137,524	1:500	1:100
Anti-MLKL	monoclonal	rabbit	Abcam, ab187091	1:1.000	1:100

Sigma Aldrich, Germany

Abcam, United Kingdom

### Immmunohistochemistry

After dewaxing and washing, paraffin-embedded sections, cut at 4-μm thickness, were incubated at RT with 0.3% hydrogen peroxide in methanol for 20 min to inactivate endogenous peroxidase. Then, the sections were washed in PBS and incubated with normal goat serum for 60 min at RT followed by RIPK1, RIPK3, CYLD or MLKL antibodies at 4 °C overnight. Specific antibodies and their dilutions were described in Table 2. After washing twice, the sections were incubated with biotinylated anti-rabbit IgG (1:500; #PK-6101, Vector Laboratories, CA, USA) for 60 min at RT. The reaction sites were visualized using 3,3 Diaminobenzidine tetrahydrochloride, TLC approx. 97% (DAB) (#D5637, Sigma Aldrich, Germany) for 5 min. The sections were counterstained for 2 min with hematoxylin. Positive immunohistochemistry staining was assessed as a characteristic brown staining using a light microscope (Zeiss Imager. Z1; Zeiss, Germany). For negative controls, primary antibodies were excluded and samples were incubated with rabbit IgG. All signals were visualized by Zeiss Axio Observer System (Carl Zeiss, Germany) using 25/0.8 NA or 63/1.3 NA immerse objectives. Images were converted to 16-bit, grayscale version and analyzed using by Zeiss Axio Observer System (Carl Zeiss, Germany) software. For quantification arithmetic means of all intensities of immunostaining were measured as optical desnity and evaluated in correlation to the signal of the control group. All values are expressed as an *n*-fold increase.

### Statistical analysis

The statistical analyses of the results of mRNA expression (n = 48 samples) and protein concentration and intensity (n = 48 samples) were performed using two way ANOVA followed by Sidak multiple comparison test (GraphPad Prism ver. 8.2.1; Graph Pad Software, San Diego, CA, USA). All numerical data are shown as standard errors of the means (± SEM) for values obtained in our experiment (six samples/treatment), each performed in duplicates. Letters ^a,b,c^ indicate statistical differences between treatment in the early and middle- stage CL. Asteriks ^*^ indicate statistical differences between local/systemic PGF_2α_ injected early CL vs local/systemic PGF_2α_ injected middle-stage CL.

## Supplementary information

**Additional file 1.** Representative western blots bands for (a) CASP3 (32 kDa), CASP8 (18 kDa), BCL2 (26 kDa), BAX (74 kDa) and ACTB (43 kDa) (b) RIPK1 (74 kDa), RIPK3 (57 kDa), CYLD (107 kDa), MLKL (54 kDa) and ACTB (43 kDa) in the early CL at 4 h after local or systemic PGF_2α_ administration; C – control group, PGF_2α_ − experimental group respectively local (2.5 mg Dinoprost intra-CL) or systemic PGF_2α_ injection (25 mg Dinoprost i.m.)

**Additional file 2.** Representative western blots bands for (a) CASP3 (32 kDa), CASP8 (18 kDa), BCL2 (26 kDa), BAX (74 kDa) and ACTB (43 kDa) (b) RIPK1 (74 kDa), RIPK3 (57 kDa), CYLD (107 kDa), MLKL (54 kDa) and ACTB (43 kDa) in the mid-stage CL at 4 h after local or systemic PGF_2α_ administrations; C – control group, PGF_2α_ − experimental group respectively local (2.5 mg Dinoprost intra-CL) or systemic PGF_2α_ injection (25 mg Dinoprost i.m.).

## Data Availability

The datasets used and analysed during the current study are available from the corresponding author on reasonable request.

## Abbreviations

aPGF_2α_: Prostaglandin F2α analogue
ANOVA: Analysis of variance
BAX: BCL2 associated X
BCL2: B-cell lymphoma 2
BCS: Body condition score
BHV1: Bovine Herpesvirus Type 1
BVD/MD: Bovine Viral Diarrhea-Mucosal Disease
CASP3: Caspase 3
CASP8: Caspase 8
cDNA: Complementary DNA
CL: Corpus luteum
CYLD: cylindromatosis
DAB: 3,3 Diaminobenzidine tetrahydrochloride
DUB: K63-specific deubiquitinating enzyme
EBL: Enzootic bovine leukosis
EDN1: Endothelin 1
FASLG: Fas ligand
i.m.: intra muscular
IFNG: Interferon gamma
LEC: Luteal endothelial cells
LSC: Luteal steroidogenic cells
MLKL: mixed lineage kinase domain-like
mRNA: messenger RNA
NO: Nitric oxide
P_4_: Progesterone
PBS: Phosphate-buffered saline
PCR: Polymerase chain reaction
PGF_2α_: Prostaglandin F2α
RIA: Radioimmunoassay
RIPK1: receptor-interacting protein kinase 1
RIPK3: receptor-interacting protein kinase 3
RT: Room temperature
TMR: Total mix ration
TNF: Tumor necrosis factor α
USG: ultrasonographic visualization
