# Supplementary figures and images for: Effects of prostaglandin F2α (PGF2α) on cell-death pathways in the bovine corpus luteum (CL)
Source: BMC Vet Res. 2019 Nov 21;15:416. doi: 10.1186/s12917-019-2167-3 (PMC6873574; doi:10.1186/s12917-019-2167-3)

A

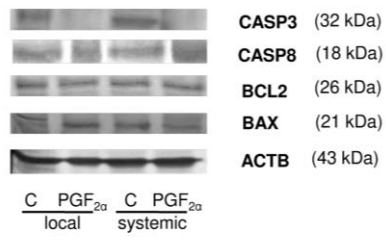

B

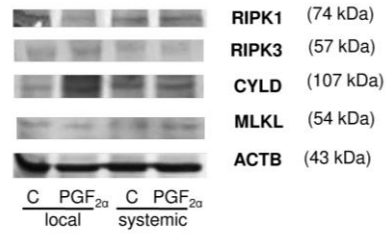

Supplement: Supplementary file 1 — Additional file 1. Representative western blots bands for (a) CASP3 (32 kDa), CASP8 (18 kDa), BCL2 (26 kDa), BAX (74 kDa) and ACTB (43 kDa) (b) RIPK1 (74 kDa), RIPK3 (57 kDa), CYLD (107 kDa), MLKL (54 kDa) and ACTB (43 kDa) in the early CL at 4 h after local or systemic PGF2α administration; C – control group, PGF2α − experimental group respectively local (2.5 mg Dinoprost intra-CL) or systemic PGF2α injection (25 mg Dinoprost i.m.) [file 12917_2019_2167_MOESM1_ESM.pdf]

A

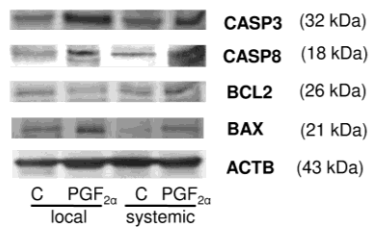

B

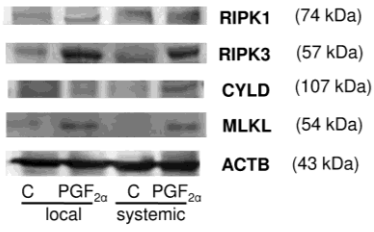

Supplement: Supplementary file 2 — Additional file 2. Representative western blots bands for (a) CASP3 (32 kDa), CASP8 (18 kDa), BCL2 (26 kDa), BAX (74 kDa) and ACTB (43 kDa) (b) RIPK1 (74 kDa), RIPK3 (57 kDa), CYLD (107 kDa), MLKL (54 kDa) and ACTB (43 kDa) in the mid-stage CL at 4 h after local or systemic PGF2α administrations; C – control group, PGF2α − experimental group respectively local (2.5 mg Dinoprost intra-CL) or systemic PGF2α injection (25 mg Dinoprost i.m.). [file 12917_2019_2167_MOESM2_ESM.pdf]
